# A PPARγ, NF-κB and AMPK-Dependent Mechanism May Be Involved in the Beneficial Effects of Curcumin in the Diabetic db/db Mice Liver

**DOI:** 10.3390/molecules19068289

**Published:** 2014-06-18

**Authors:** Lizbeth M. Jiménez-Flores, Sergio López-Briones, Maciste H. Macías-Cervantes, Joel Ramírez-Emiliano, Victoriano Pérez-Vázquez

**Affiliations:** Departamento de Ciencias Médicas, División de Ciencias de la Salud, Campus León, Universidad de Guanajuato. León, 37320, Mexico; E-Mails: lizmjf@gmail.com (L.M.J.-F.); lobris@yahoo.com (S.L.-B.); macistehabacuc@yahoo.com.mx (M.H.M.-C.); joelre@ugto.mx (J.R.-E.)

**Keywords:** antioxidants, *Curcuma longa*, diabetes, inflammation, obesity, polyphenols

## Abstract

Turmeric (*Curcuma longa*) is a rhizomatous herbaceous perennial plant of the ginger family which has been used to treat biliary disorders, anorexia, cough, rheumatism, cancer, sinusitis, hepatic disorders, hyperglycemia, obesity, and diabetes in both Ayurvedic and Traditional Chinese Medicine. Suggested mechanisms of action include the modulation of signal transduction cascades and effects on gene expression, however they remain to be elucidated. In this study, the expression of some proteins responsible for transcription factors, inflammation, and metabolic control were evaluated by western blot in 15-week-old db/db mice livers treated with curcumin 0.75% mixed in their diet for 8 weeks. In addition, nitrosative stress was evaluated. Curcumin increased the expression of AMPK and PPARγ, and diminished NF-κB protein in db/db mice. However, it did not modify the expression of PGC-1α or SIRT1. Nitrosative stress present in db/db mice livers was determined by a unique nitrotyrosylated protein band (75 kDa) and was not reverted with curcumin. In conclusion, curcumin regulates the expression of AMPK, PPARγ, and NF-κB; suggesting a beneficial effect for treatment of T2DM complications. In order to observe best beneficial effects it is desirable to administer curcumin in the earlier states of T2DM.

## 1. Introduction

Curcumin is a natural yellow polyphenol extracted from the rhizome of turmeric (*Curcuma longa*), which is extensively used as a spice in food preparation in Asian countries. Curcumin has been shown to have a wide range of pharmacological properties and it has been used to treat biliary disorders, anorexia, cough, rheumatism, cancer, sinusitis, hepatic disorders, and antiviral, antioxidant and anti-inflammatory effects. However, the molecular network that governs curcumin-derived effects on health has not been completely elucidated [1].

A number of excellent reviews exist that summarize the multiple molecular targets and beneficial effects of curcumin on different biological systems and animal models [2,3,4]. Beneficial effects of curcumin on metabolic syndrome, obesity, diabetes, and other complications derived from these [5,6] have been pointed out, with whole book chapters written on the many beneficial properties of curcumin [7]. It is also well documented that curcumin could be potentially used in the treatment of diabetes, and the complications that arise from it, primarily because it is a relatively safe and inexpensive drug [8].

Type 2 diabetes mellitus (T2DM) is a common metabolic disease with a high and growing prevalence; it affected 382 million people worldwide as of the year 2013 and is expected to affect 592 million by 2035 [9]. T2DM is characterized by a chronic state of hyperglycemia, which induces an intracellular elevation of reactive oxygen species (ROS). The cumulative ROS can consequently cause long-term dysfunctions in different organs such as the liver [10]. Curcumin possesses certain properties that could alleviate liver damage or hepatic dysfunction problems associated with T2DM [11].

Previous studies showed that curcumin has a protective role against oxidative stress and mitochondrial dysfunction in the kidneys and in the liver of high-fat diet (HFD)-induced obese mice, through the increase of oxygen consumption and the decrease of lipid and protein oxidation levels [12]. Moreover, evaluation of curcumin’s effect in the hippocampus and frontal cortex of diabetic db/db mice and in the sera of obese humans, showed that curcumin decreases obesity-induced protein oxidation in humans and restores brain-derived neurotrophic factor (BDNF) levels in mice [13]. 

Obesity and T2DM are associated with low-grade chronic inflammation, in part due to the activation of the nuclear factor kappa-B (NF-κB). The anti-inflammatory pathways of curcumin have been described mostly *in vitro* from tumor cell studies, attained mainly through the down-regulation of NF-κB [14]. In turn, NF-κB deregulates the activity of other metabolic pathways where Sirtuin 1 (SIRT1) and the AMP-activated protein kinase (AMPK) are involved; these are regulatory proteins of certain intracellular routes, such as glucose uptake in skeletal muscle and hepatic fatty acid oxidation. In 3T3-L1 adipocyte cell culture systems, curcumin increased AMPK activity by inducing the phosphorylation of AMPK, thereby improving the lipid metabolism in adipocytes; this suggests that curcumin has a potential benefit in preventing obesity [15]. On the other hand, some reports suggest that curcumin also activates SIRT1 [16]. In the liver, SIRT1 participates in the control of glucose homeostasis, through deacetylation of the peroxisome proliferator-activated receptor-gamma coactivator 1 alpha (PGC-1α) and inducing the downstream target genes, such as the peroxisome proliferator-activated receptor gamma (PPARγ) gene. Consequently, PGC-1α and PPARγ coordinate the transcription of metabolic genes; increasing the hepatic glucose production and use of fatty acids for energy production [17,18]. The curcumin effects on the PGC-1α expression and activity in obesity or T2DM have not been completely clarified.

Antioxidant effects of curcumin may reflect prevention of T2DM-induced post-translational modification events involving nitration of tyrosine residues to yield nitrotyrosine (NT) in proteins. However, few studies are available regarding the specific liver proteins that are targets of nitration during T2DM [19]. The present study was designed to fully characterize and investigate the effects of curcumin on (a) NF-κB expression as a key factor of inflammation; (b) AMPK and SIRT1 expression as metabolic enzymes related to caloric restriction and energetic sense; (c) PGC-1α and PPARγ, as transcription regulators of genes related to carbohydrates metabolism, fatty acids oxidation, and mitochondrial biogenesis, and (d) the nitrosative state in diabetic db/db mice liver.

## 2. Results and Discussion

### 2.1. Effect of Curcumin on Body Weight and Blood Glucose Levels

**Figure 1:**
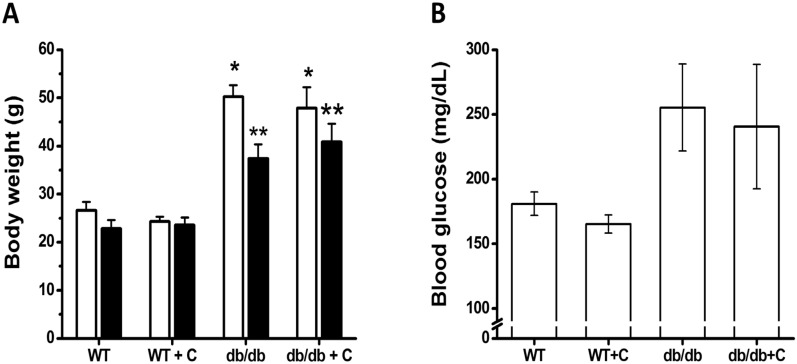
Effects of curcumin on body weight and blood glucose levels of mice. (**A**) Body weight: The body weight of mice was measured at the beginning and end of curcumin treatment. (**B**) Blood glucose levels: The levels of glucose were determined at the end of curcumin treatment. □, initial weight. ■, final weight. Data are the means of *n* = 3 ± SEM. * *vs.* WT and WT+C (initial), *p* < 0.001; ** *vs.* WT and WT+C (final), *p* < 0.01.

Mice were weighed at the beginning of the curcumin treatment; at this point no significant differences were observed between the groups that were named wild type (WT). WT were untreated or treated with 0.75% curcumin (WT+C) while diabetic groups were (db/db) and db/db treated with 0.75% curcumin (db/db+C) (24.2 ± 0.8, 25.2 ± 0.6, 46.9 ± 4.9 and 47.9 ± 4.3 g, respectively; Figure 1A). The groups of diabetic mice were heavier than the groups of WT mice (*p* < 0.001).

As shown in Figure 1A, at the end of curcumin treatment, the db/db and db/db+C groups (34.6 ± 7.0 and 40.8 ± 6.5 g, respectively) were statistically weighted (*p* < 0.01) over the WT and WT+C groups (22.8 ± 2.5 and 23.6 ± 3.9 g, respectively). Interestingly, during the treatment period, the db/db mice lost more weight (12.5 ± 3.0 g) than db/db+C mice (7.0 ± 1.3 g); however this difference was not statistically significant. The same effect was reported by Seo *et al.* [20]; a 6 week-curcumin treatment suppressed body weight loss in db/db mice. In prior research, has been observed that changing the diet of male mice at 7 weeks to include curcumin results in weight gain after 8 weeks [13]. In the present study, 15 week-old mice were used, at an age at which hyperglycaemia causes serious metabolic alterations that are reflected in accelerated weight loss [21]. Unfortunately in this study, curcumin failed to prevent accelerated weight loss in diabetic mice compared with untreated-diabetic mice. However, it is desirable to administer curcumin in earlier states of T2DM in order to observe best beneficial effects on body weight. As shown in Figure 1B, curcumin treatment did not have any effect (*p* = 0.07) on blood glucose levels in db/db mice. Blood glucose levels in db/db and db/db+C mice were higher (255.4 ± 33.6 and 240.6 ± 48.1 mg/dL, respectively) than in WT and WT+C mice (181 ± 9.1 and 165.2 ± 6.9 mg/dL, respectively). These results confirm that the db/db mice were diabetic and obese, which is similar to previously reported results [13]. In the past, several studies, have reported that curcumin reduces blood glucose levels in db/db mice [13,20]. Nonetheless, in 7 week-old high-fat diet (HFD)-induced obese mice was not observed any hypoglycemic effect from curcumin [12]. Another study reported that dietary curcumin admixture ameliorated diabetes in (HFD)-induced obese and ob/ob mice, as determined by glucose and insulin tolerance testing [22]. Additionally, it was observed that short-term curcumin gavage improved glucose disposal in a HFD-induced mouse model in the absence of an apparent change of body weight [23]. In humans, has been demonstrated that curcumin improvements diabetic retinopathy, although blood pressure, heart rate and diabetic control were unchanged [24]. One reason why, in the present study, we did not find any blood glucose concentration changes in diabetic mice may be due to advanced complications of diabetes such as marked hypoinsulinemia and β cell pancreatic destruction reported to db/db mice in 15–23 weeks of age [25], suggesting that curcumina administration was late. Further studies are required to establish a potential metabolic regulator activity of curcumin.

### 2.2. Effect of Curcumin on NF-κB Expression

**Figure 2:**
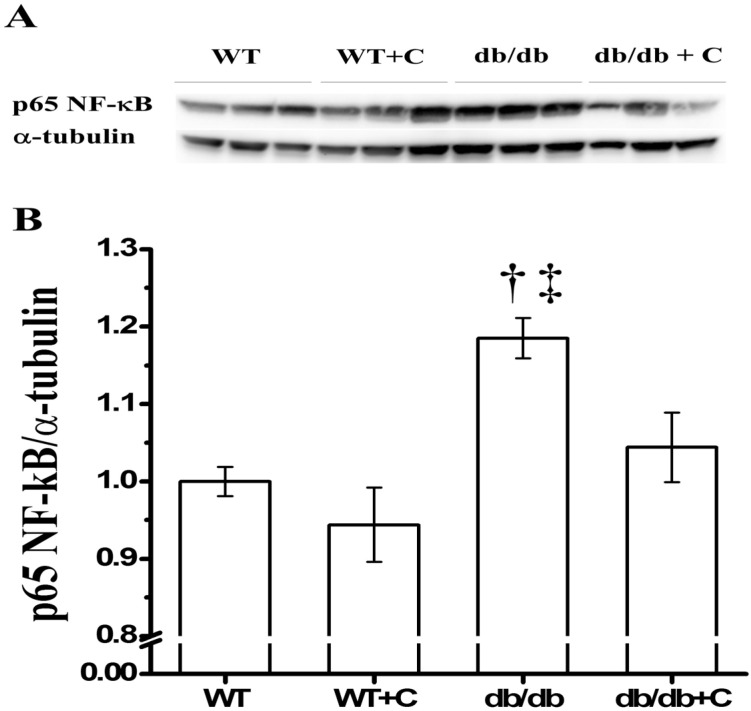
Effect of curcumin on NF-κB expression in liver. (**A**) Representative western blot of the NF-κB and α-tubulin. (**B**) Densitometry analysis of the NF-κB/α-tubulin ratio; ^†^ *vs.* WT and db/db+C, *p* < 0.05; ^‡^ *vs.* WT+C, *p* < 0.005.

Transcription factor NF-κB is a key protein that regulates the inflammatory response. As shown in Figure 2, when compared with the WT and WT+C mice (1.0 ± 0.019 and 0.944 ± 0.048, respectively) there was an increase of the NF-κB expression in db/db mice (1.185 ± 0.026), while curcumin significantly attenuated this effect in db/db+C mice (1.044 ± 0.045) compared with untreated db/db mice. This result suggests NF-κB diminution underlies the curcumin potential as an anti-inflammatory agent in diabetes, according to inflammation, obesity, and insulin-resistance reports [26,27], e.g., in humans, curcumin administration improves diabetic microangiopathy; a process related to the inflammatory effect of NF-κB activation [24].

Other research teams have reported that curcumin decreases the expression and nuclear activity of NF-κB in different tissues and human myeloid ML-1a cells [14]. Thus, the decreased NF-κB expression observed in the present study is important, because NF-κB is a key factor that triggers and increases the chronic inflammatory response related to different chronic degenerative diseases and its complications. NF-κB would thus be an important therapeutic target of curcumin for the control of chronic inflammation in obesity and diabetes.

### 2.3. Effect of Curcumin on AMPK and SIRT1 Expression

**Figure 3:**
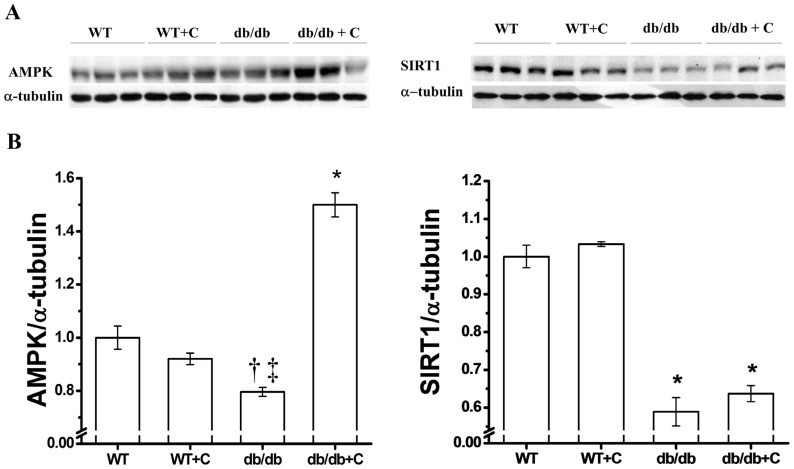
Effect of curcumin on AMPK and SIRT1 expression in liver. (**A**) Representative western blot of the AMPK and SIRT1 expression (**B**) Densitometry analysis of the AMPK/α-tubulin and SIRT1/α-tubulin ratios. * AMPK *vs.* WT, WT+C y db/db, *p* < 0.001; **^†^** *vs.* WT+C, *p* < 0.05; **^‡^** *vs.* WT, *p* < 0.005; * SIRT1 *vs.* WT y WT+C, *p* < 0.001.

There seems to be a link between metabolic syndrome and AMPK signal pathways, so we decided to determine the effect of curcumin on AMPK expression levels in diabetic mice livers. As shown in Figure 3A, the protein expression is significantly reduced in db/db mice (0.796 ± 0.017) compared with the two healthy groups (1.0 ± 0.044 and 0.92 ± 0.022) and curcumin increased the expression around 50% in diabetic mice (1.5 ± 0.045) when compared to the WT group (*p* < 0.001).

In rodent models of obesity, a decrease of AMPK in peripheral tissue such as in the heart, skeletal muscle, and liver has been observed [28]. It has been demonstrated that resveratrol stimulates AMPK activity in several cell types [29]. The increased AMPK expression in diabetic mice is important since AMPK inhibits hepatic glucose production and lipogenesis; these processes that are affected in obesity and T2DM [30,31]. Also, AMPK activation is linked to SIRT1, that is to say SIRT1 functions as a novel upstream regulator for LKB1/AMPK signaling and plays an essential role in the regulation of hepatocyte lipid metabolism [32,33].

Figure 3B shows that the expression of SIRT1 was decreased in db/db mice (0.589 ± 0.037), which was in contrast to the WT and WT+C groups (1.0 ± 0.03 and 1.033 ± 0.006, respectively). Curcumin did not have any significant change on the SIRT1 expression in db/db+C mice (0.637 ± 0.021, Figure 3B). SIRT1 is a NAD^+^ dependent histone deacetylase that is induced by caloric restriction; in the liver it regulates the glucose/lipid metabolism, glucose production, and fatty acid oxidation through its deacetylase activity on many substrates. Thus, SIRT1 is an important regulator of energy metabolism. Recent studies have demonstrated that SIRT1 is down-regulated in several cells and tissues in insulin-resistant or glucose intolerance states [34,35,36]. The decreased SIRT1 expression observed in the present study may contribute to the development of T2DM-related dysfunctions, therefore SIRT1 activation may be a candidate therapeutic target for T2DM. However, it has been suggested that curcumin, as resveratrol, exerts an indirect activation of SIRT1 protein through AMPK [37]. The functions of SIRT1 are varied and tissue-specific, an example of this is that SIRT 1 can be toxic or beneficial dependent of cellular type in brain [38] by regulation of gene transcription of multiple metabolic and signaling pathways. As an example, SIRT1 regulates the transcriptional activity of transcription factors like PPARγ through deacetylation and further activation of its coactivator PGC-1α [39,40].

### 2.4. Effect of Curcumin on PGC-1α and PPARγ Expression

**Figure 4:**
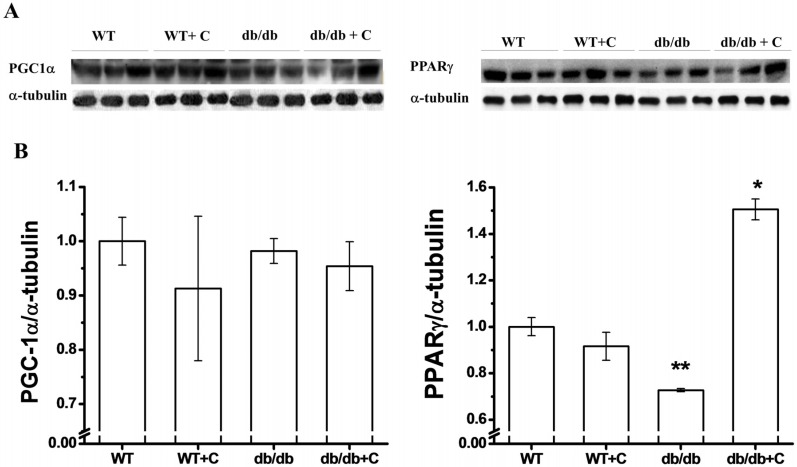
Effect of curcumin on PGC-1α and PPARγ expression in liver. (**A**) Representative western blot of the PGC-1α and PPARγ expression; (**B**) Densitometry analysis of the PGC-1α/α-tubulin and PPARγ/α-tubulin ratios. Data are the means of *n* = 3 ± SEM. * *vs.* WT, WT+C and db/db, *p* < 0.001; ** *vs.* WT, *p* < 0.01.

In regard to PGC-1α expression (Figure 4A), we did not observe any difference between the WT and WT+C mice (1.0 ± 0.04 and 0.91 ± 0.13, respectively) and the two groups of db/db and db/db+C mice (0.98 ±0.02 and 0.95 ± 0.04, respectively). Puigserver and Spiegelman [41] reported that in mice liver, the PGC-1α expression is increased with type 1 and type 2 diabetes. However in other tissues, such as skeletal muscle, there are reports of a decrease of the PGC-1α expression in both diabetic subjects and family-history-positive nondiabetic subjects [42]. Interestingly, in the present study no difference was observed among the four groups of mice; curcumin did not have any effect on the PGC-1α expression in the db/db group. In one of few reports on increasing PGC-1α, it is suggested that the effects of this protein are mediated by increasing the PGC-1α activity rather than by increasing the PGC-1α protein expression [43].

As shown in Figure 4B, the expression of PPARγ was lower in the db/db group (0.727 ± 0.007) as compared to the WT group (*p* < 0.01). The curcumin treatment reversed that reduction in db/db mice (1.505 ± 0.045) as compared with the three other groups (*p* < 0.001). PPARγ belongs to a protein-regulated SIRT1 category, which is a regulator of glucose/lipid metabolism in adipose tissue, skeletal muscle, heart, and liver [17]. The decrease of the PPARγ expression in diabetic mice that we observed is in accordance with the above, whereas hepatic glucose metabolism is altered in the diabetic condition [44]. Therefore, PPARγ is considered as a therapeutic target for T2DM treatment. Three studies independently demonstrated that the level of PPARγ and its *trans*-activating activity were diminished during hepatic stellate cells (HSC) activation *in vitro*, whereas NF-κB activity was increased [45,46,47]. In the present study, increased NF-κB expression was observed, while the PPARγ expression was decreased in non-supplemented db/db mice. The effect that curcumin exerts on PPARγ is not fully understood but some studies have shown that curcumin inhibits HSC activation by increasing the activity of PPARγ [48,49]. This could be an important beneficial effect of curcumin, similar to other drugs like Thiazolidinediones (TZDs) that increase the PPARγ expression; with the advantage that curcumin does not have side effects [50] which is supported by curcumin’s safe track record, demonstrated in several studies [8]. Therefore, curcumin is a potential antifibrotic candidate for treatment of hepatic fibrogenesis and an antioxidant agent for T2DM prevention.

Transcription coactivator PGC-1α regulates the coordinated expression of several proteins such as PPARγ. It is supposed that PGC-1α, once activated, mediates the activation of PPARγ by docking on, and co-activating, transcription factors that control expression of gene of PPARγ in a similar way to what was suggested in skeletal muscle to other proteins [43].

### 2.5. Effect of Curcumin on the Protein Nitration Content

**Figure 5:**
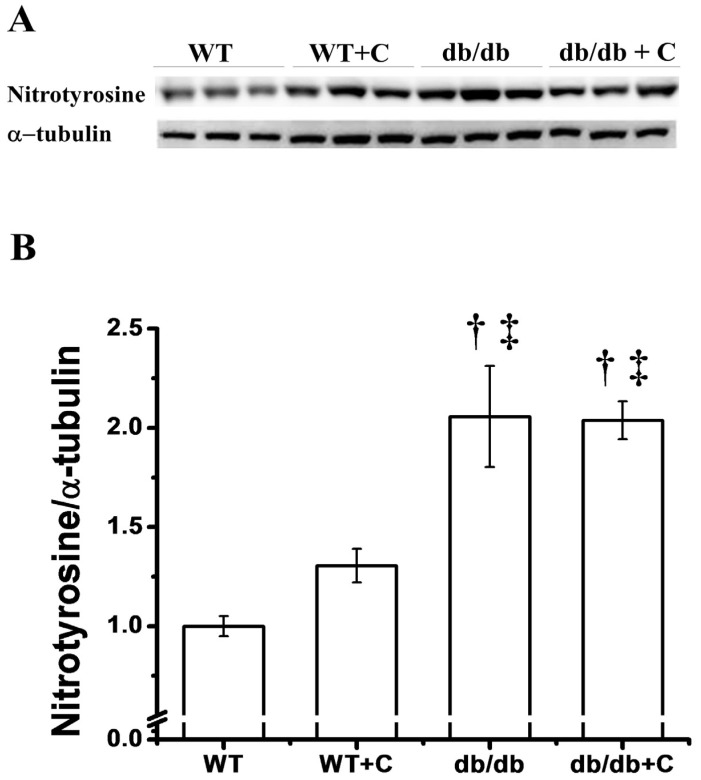
Effect of curcumin on the protein nitration (NT) content. (**A**) Representative western blot of the NT and of α-tubulin in liver. (**B**) Densitometry analysis of the NT/α-tubulin ratio. Data are the means of *n* = 3 ± SEM. ^†^ *vs.* WT+C, *p* < 0.05; ^‡^ *vs.* WT, *p* < 0.005.

Interestingly, we only observed one band of nitrotyrosilated protein at approximately 75 kD. Figure 5 shows that in db/db mice the NT content is elevated (2.05 ± 0.255) when compared to WT and WT+C mice (1.0 ± 0.051 and 1.305 ± 0.085, respectively).

In this study, curcumin treatment did not have a significant effect to ameliorate the NT abundance in db/db+C mice (2.038 ± 0.095). Nitrosative stress plays a key role in damage to proteins, leading to a series of mitochondrial proteins nitration in diabetic patients. One study reported that in streptozotocin-induced diabetic rats, increased levels of inducible nitric oxide synthase (iNOS) mRNA and NT content were observed, in accordance with pathological alterations of the ultrastructure of liver mitochondria [19]. Ren *et al.* [19] reported that treatment with aminoguanidine could significantly reduce the content of oxidative stress-induced nitrated mitochondrial proteins. In this study, curcumin treatment did not ameliorate the NT content in diabetic mice.

A more recent study reported that enalapril, an angiotensin-converting enzyme inhibitor, blunted the T2DM-induced increase in tyrosine nitration of three mitochondrial proteins of the renal cortex by a mechanism involving suppression of oxidant production and enhancement of antioxidant capacity [51]. No information is available regarding the curcumin activity against protein nitration in liver; however, one study did demonstrate the antioxidant properties of curcumin and its protective effects against oxidative/nitrative changes of blood platelets and plasma components, especially proteins and lipids by preventing partial 3-nitrotyrosine formation in human plasma proteins [52]. Additionally, the glutamoyl diester, a bioconjugate of curcumin, protected against oxidative stress-dependent brain mitochondrial complex inhibition and protein nitration compared to curcumin alone, suggesting a neuroprotective activity of diglutamoyl curcumin against neurodegenerative diseases [53]. The lack of effect of curcumin on the content of the nitrated protein could be due to the advanced state of diabetes, suggesting the need for more studies in earlier states of diabetes to confirm, or exclude, the beneficial effect of curcumin on reduction in content of nitrated proteins in diabetic mouse liver. Probably this antioxidant have a preventive function, for this reason it will be important to use curcumin in earlier states of T2DM, before nitrosative stress is established.

## 3. Experimental

### 3.1. Reagents and Antibodies

Sucrose, potassium chloride, sodium chloride, ethylenediamine tetraacetic acid (EDTA), polyvinilpirrolidone (PVP), and phenol were purchased from Sigma Chemical (St. Louis, MO, USA). Tris, sodium dodecyl sulfate (SDS), tween-20, glycerol, glycine, and 2β-mercaptoethanol were purchased from Bio-Rad (Mexico City, Mexico). Complete tablet (a protease inhibitor) was obtained from Roche Diagnostics (Mannheim, Germany). Rabbit polyclonal antibodies against mouse PGC-1α (sc-13067), PPARγ (sc-7196), SIRT1 (sc-74465), AMPKα1/2 (sc-25792), p65 NF-κB (sc-33039), and mouse monoclonal antibody against 3-Nitrotyrosine (sc-32757), and α-Tubulin (sc-32293), such as HRP-conjugated goat anti-rabbit IgGs (sc-2004) and anti-mouse IgG1 (sc-2060) were obtained from Santa Cruz Biotechnology (Palo alto, CA, USA). Curcumin was purchased from Advanced Nutrition (Guadalajara, Jalisco, Mexico).

### 3.2. Animals and Treatment

Fifteen-week-old male mice were treated as follows: three diabetic *db/db* (BKS.Cg- Leprdb/Leprdb/OlasHsd) and three wild-type mice were fed a standard diet (Cat. No. T.2018S.15, Harlan Laboratories, Mexico City, Mexico) supplemented with 0.75% of curcumin for a period of 8 weeks; 3 db/db and 3 wild-type mice were used as controls. All mice had access to water and food *ad libitum*. All animal procedures were performed in accordance with current Mexican legislations (NOM-062-ZOO-1999) and according to the National Institutes of Health’s (Bethesda, MD, USA) Guide for Care and Use of Laboratory Animals.

At the end of treatment, mice were killed by decapitation. Then levels of blood glucose were determined using a commercial kit (Accutrend GCT, Roche, Mexico City, Mexico).

### 3.3. Liver Proteins Extraction

Liver proteins were obtained by homogenization at 4 °C in a potter in the presence of a 106-protease inhibitor (Complete tablets; Roche Diagnostics GmbH, Mannheim, Germany). To further limit proteolysis, protein isolation was performed using phenol extraction as described in [54]. To solubilize and obtain completely denatured and reduced proteins, pellets were dried and resuspended in Laemli buffer (Tris-HCl 0.125M, SDS 4%, Glycerol 20%, 2β-mercaptoethanol 10%, pH 6.8) as previously reported [55]. To determinate protein concentration, the modified Lowry procedure was used [56] (DC Protein Assay, Bio-Rad, Mexico City, Mexico).

### 3.4. Western Immunobloting Assay

The total proteins were separated by 12% SDS-PAGE and transferred to a VPDF membrane in a Mini Trans-Blot cell (Bio-Rad) for 1 h at 100 V. The VPDF membranes were blocked with Tris-buffered saline and Tween-20 (TBST: 25 mM Tris, 150 mM NaCl, 0.1% Tween-20, pH 7.6) containing 2% nonfat dry milk at room temperature for 3 h. The separated proteins were probed with a specific antibody against PGC-1α (1:1,000), PPARγ (1:500), SIRT1 (1:500), AMPKα1/2 (1:1,000), p65 NF-κB (1:1,000), Nitrotyrosine (1:500), and α-Tubulin (1:3,000) overnight at 4 °C. After four washes with TBST, the complexes were detected by HRP-conjugated goat anti-rabbit Ig antibodies using an enhanced chemiluminescence protein detection kit (Wester Lightninh™ Plus-ECL, Perkin Elmer, Waltham, MA, USA). The results were normalized according to α-Tubulin values. Densitometric analyses were conducted using Image Lab Software (Bio-Rad).

### 3.5. Statistical Analysis

All results were presented as mean ± standard error of the mean (SEM) from at least three independent experiments. The results were assessed by a Kruskal Wallis test. In addition, a *post hoc* Tukey’s test was performed for multiple comparisons among different groups. Differences with the *p* value less than 0.05 were considered statistically significant. Statistical analysis was performed using STATISTICA 10 Software (StatSoft^®^, Tulsa, Oklahoma, USA).

## 4. Conclusions

Curcumin regulates the expression of AMPK, PPARγ, and NF-κB, but not of SIRT1 and PGC-1α in the liver of db/db mice; however, these proteins could be activated as discussed prior in the results and discussion section. The study of molecules involved in the disturbance of metabolic pathways may be employed in order to direct appropriate therapeutic strategies for T2DM complications. This study provides a basis to elucidate a possible molecular mechanism of action of curcumin in the liver. The therapeutic potential of curcumin is constrained by limited bioavailability; this could be a relative disadvantage in regards to beneficial curcumin effects. At the age of 15 weeks, the curcumin intervention on db/db mice could be too late to allow a major reversal of complications or improvement from the disease. Further studies are needed to define the ideal timing and procedure to use curcumin in T2DM.
